# Desfechos após Parada Cardiorrespiratória Extra-Hospitalar de Natureza Clínica e Traumática

**DOI:** 10.36660/abc.20220551

**Published:** 2023-07-19

**Authors:** Daiana Terra Nacer, Regina Márcia Cardoso de Sousa, Anna Leticia Miranda

**Affiliations:** 1 Universidade de São Paulo Escola de Enfermagem São Paulo SP Brasil Universidade de São Paulo – Escola de Enfermagem, São Paulo, SP – Brasil; 2 Universidade Federal de Minas Gerais Faculdade de Medicina Belo Horizonte MG Brasil Universidade Federal de Minas Gerais – Faculdade de Medicina – Campus Saúde, Belo Horizonte, MG – Brasil

**Keywords:** Parada Cardíaca, Parada Cardíaca Extra-Hospitalar, Reanimação Cardiopulmonar, Serviços Médicos de Emergência, Sobrevivência

## Abstract

**Fundamento:**

Dados sobre Parada Cardiorrespiratória extra-hospitalar ainda são escassos, muito variados e indicam mau prognóstico para eventos traumáticos.

**Objetivos:**

Descrever a sobrevivência extra/intra-hospitalar, o tempo de sobrevivência e as condições neurológicas dos atendidos por unidades de suporte avançado à vida e submetidos a ressuscitação cardiopulmonar e comparar os resultados das paradas cardiorrespiratórias de natureza clínica e traumática.

**Métodos:**

Estudo de coorte, realizado em três etapas, nas duas primeiras, os dados foram coletados em fichas do Serviço de Atendimento Móvel de Urgências e prontuários, na terceira, foi aplicada a Escala de Categoria de Performance Cerebral. A casuística foi de vítimas reanimadas com idade ≥18 anos. Os testes de Fisher e log-rank foram empregados na comparação das causas, considerando nível de significância de 5%.

**Resultados:**

Foram analisados 852 pacientes, 20,66% foram hospitalizados, 4,23% sobreviveram até transferência ou alta, 58,33% apresentaram desfecho favorável um ano após parada. Houve associação entre sobrevivência pré/intra-hospitalar e natureza da ocorrência (p=0,026), porém não houve diferença entre as curvas de sobrevivência, p=0,6.

**Conclusões:**

A sobrevivência à hospitalização após parada cardiorrespiratória extra-hospitalar foi baixa, porém, a maioria dos sobreviventes à alta alcançaram desfecho favorável após um ano. O tempo de sobrevivência dos hospitalizados após eventos de natureza clínica e traumática foram similares, porém a sobrevida pré-hospitalar foi maior entre os traumatizados.

**Figura Central:**
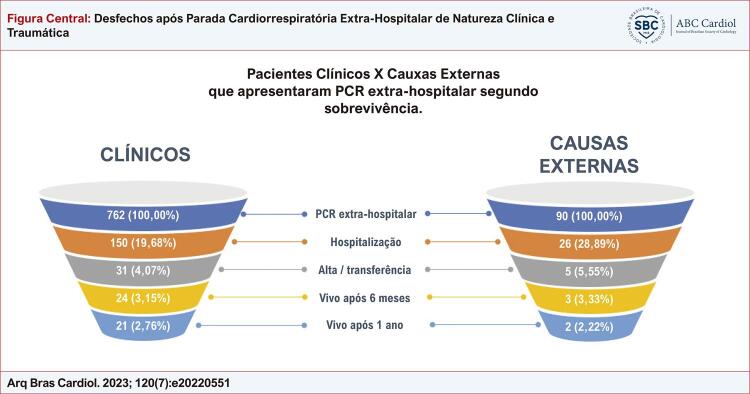
: Desfechos após Parada Cardiorrespiratória Extra-Hospitalar de Natureza Clínica e Traumática

## Introdução

A parada cardiorrespiratória (PCR) é a perda abrupta da função cardíaca.^1^ É um evento de alta prevalência, com morbidade e mortalidade elevadas.^2^

Apesar dos avanços no atendimento, a sobrevida desses eventos é baixa, principalmente em ambiente extra-hospitalar. Dados da literatura ainda são escassos no Brasil, muito variados ao redor do mundo e indicam mau prognóstico para a PCR de natureza traumática.^2^

Segundo a Sociedade Brasileira de Cardiologia, a sobrevida nas PCR de natureza traumática é em torno de 0 a 2,6%, sendo os esforços de ressuscitação cardiopulmonar (RCP) considerados estéreis em muitos estudos.^2^

As diretrizes europeias de ressuscitação de 2015 estimulam novos estudos sobre PCR de natureza traumática, visto que existe uma variação considerável na sobrevida relatada na literatura científica (intervalo de 0 a 27%), o que reflete a heterogeneidade nos casos relatados e a desigual assistência prestada em diferentes sistemas.^3^

Em geral, as estatísticas atuais sobre PCR extra-hospitalar demonstram importantes variações geográficas quanto à desfechos desses eventos. Chamam atenção alguns locais com resultados muito ruins e outros que alcançam importantes frequências de sobrevida, prováveis consequências de esforços para otimizar a eficácia da cadeia local de sobrevivência, obtida pela identificação e ajustes de seus elos fracos.^4^

Nas análises da qualidade da assistência à PCR, diferentes desfechos têm sido valorizados, o retorno à circulação espontânea (RCE), a sobrevida até a hospitalização e até a alta hospitalar e a condição neurológica a curto e médio prazos. Nos pacientes que têm RCE após uma PCR, a recuperação de uma lesão cerebral anóxica é variável e uma gama de sequelas neurológicas pode acontecer, desde a recuperação completa até o coma com morte cerebral. Assim, idealmente, a avaliação do desfecho da PCR deve incorporar o estado funcional e neurológico.^5^

Este estudo se justifica em virtude da relevância do tema apresentado, e pela falta de dados sobre sobrevivência e desfecho neurológico a curto e médio prazo de pessoas que apresentaram PCR extra-hospitalar. Tem como objetivos: Descrever a sobrevivência extra/intra-hospitalar, o tempo de sobrevivência e as condições neurológicas dos atendidos por unidades de suporte avançado à vida (SAV) e submetidos a RCP e comparar os resultados das PCR de natureza clínica e traumática.

## Método

### Delineamento do estudo

Trata-se de um estudo regional, de coorte, realizado em Campo Grande, Mato Grosso do Sul (MS), Brasil e desenvolvido em três etapas. Nas duas primeiras, foi realizada uma coleta de dados retrospectiva, sendo as fontes, na primeira etapa, as fichas de atendimento pré-hospitalar (APH) e, na segunda, os prontuários dos pacientes sobreviventes do APH encaminhados para unidades hospitalares. Na terceira, os sobreviventes à internação ou seus familiares foram entrevistados. Nesta fase, a coleta foi prospectiva.

### Local de coleta de dados

Os dados da primeira etapa foram coletados no Serviço de Atendimento Móvel de Urgências (SAMU), na segunda etapa a coleta das informações foi realizada nos três hospitais que são portas hospitalares de urgência na Rede de Atenção às Urgências e Emergências. A última fase foi realizada no domicílio dos sobreviventes à internação hospitalar.

### Período

A coleta de dados foi iniciada em maio de 2018 sendo finalizada em março de 2020.

### População e critérios de seleção

A população foi composta por sujeitos com idade de 18 anos ou mais que apresentaram PCR extra-hospitalar no período de 1 de janeiro de 2016 a 31 de dezembro de 2018 e que receberam manobras de RCP pela equipe de APH de suporte avançado do SAMU (incluindo os casos em que foi iniciada por outra equipe, por leigos e outros).

Foram excluídos gestantes e pacientes com fichas ilegíveis e incompletas, isto é, que não permitiam acessar a descrição de mais de 50% das variáveis clínicas da pesquisa. Também foram excluídas as fichas dos casos transferidos para hospitais não qualificados como portas hospitalares da Rede de Atenção às Urgências e Emergências de Campo Grande.

### Instrumentos utilizados para coleta de informações

A coleta de dados foi realizada por meio do preenchimento de dois formulários, elaborados pelas pesquisadoras: o primeiro instrumento permitiu a transcrição das informações do APH, baseada nos dados disponíveis nas fichas do APH do SAV do SAMU (ficha do médico e enfermeiro). As informações do atendimento intra-hospitalar, coletadas em prontuário, também foram registradas nesse primeiro formulário. O segundo instrumento incluiu as informações sobre as condições neurológicas dos pacientes na alta, aos seis meses e um ano após PCR que foram coletadas na visita domiciliar realizada para os pacientes que sobreviveram à internação hospitalar.

Para avaliar a condição neurológica dos pacientes sobreviventes à hospitalização foi aplicada a Escala de Categoria de Performance Cerebral (CPC), conforme recomendado pela Sociedade Brasileira de Cardiologia.^6^ Este sistema de pontuação permite avaliar a capacidade funcional após PCR com base em entrevistas à família e em informação registrada, apontando os escores da CPC na alta, aos seis meses e em um ano. Os resultados foram apresentados utilizando as cinco categorias da escala: CPC 1 (Bom desempenho cerebral); CPC 2 (Incapacidade cerebral moderada); CPC 3 (Incapacidade cerebral grave); CPC 4 (Comatoso, estado vegetativo) e CPC 5 (Morte). Nas análises essas categorias também foram dicotomizadas em favorável, CPC 1 e 2, e desfavorável, CPC 3, 4 e 5.

### Coleta de dados

Na primeira fase da coleta de dados foram consultadas todas as fichas de atendimentos realizados pelas unidades de SAV do SAMU de 2016 a 2018, e as fichas referentes à PCR foram separadas manualmente. Foram excluídas fichas de menores de 18 anos, gestantes, institucionalizados e as fichas incompletas (menos de 50% das variáveis clínicas do estudo preenchidas), além das fichas dos transferidos para hospitais que não participaram da pesquisa. A informação referente aos tempos de acionamento das ambulâncias e deslocamentos foi coletada no sistema eletrônico da Central de Regulação do SAMU.

Na segunda fase, foram coletados dados dos prontuários dos pacientes encaminhados aos três hospitais da Rede de Atenção às Urgências e Emergências de Campo Grande. Nessa fase, verificou-se o desfecho da internação e aplicou-se a escala CPC com base nos registros de prontuários.

Na terceira fase, os pacientes que sobreviveram à internação hospitalar ou seus familiares foram convidados a participar do estudo por meio de ligação telefônica. Após a anuência dos participantes, foi realizada uma visita domiciliar para coleta de dados por meio de entrevista e assinatura do Termo de Consentimento Livre e Esclarecido (TCLE).

Nessa fase do estudo, todas as entrevistas foram realizadas no mínimo um ano após PCR e, para aqueles pacientes incapazes de se comunicar, foi estabelecido um responsável para fornecer as informações. O escore de CPC obtido pela análise dos prontuários foi validado durante a entrevista dessa fase da pesquisa e as informações sobre a condições neurológicas dos pacientes aos seis meses e um ano após PCR foram questionadas a fim de estabelecer os escores da CPC nesses dois últimos períodos.

### Tratamento e análise dos dados

Os dados coletados foram armazenados em banco de dados do Microsoft Office Excel^®^, versão 2016 e esse programa também foi utilizado para realizar as análises descritivas. As provas estatísticas foram realizadas segundo orientação de profissional da área e para sua realização foi aplicado o pacote estatístico R, versão 4.1.0, considerando nível de significância de 5%.

Variáveis categóricas foram descritas por meio de frequências absolutas e relativas, as variáveis contínuas foram apresentadas de forma intervalar e a média e o desvio padrão (DP) foram calculados nos casos de distribuição de dados normal.

Na comparação dos desfechos das vítimas de PCR de causa clínica e traumática foram analisadas como variáveis dependentes a condição vital das vítimas até a hospitalização e alta hospitalar (variáveis categóricas), além do tempo de sobrevida em dias após PCR (variável contínua). A natureza da PCR (clínica ou traumática) foi uma variável independente categórica para essas análises.

Para avaliar associação entre variáveis categóricas foram aplicados os testes Qui-quadrado de Pearson e Exato de Fisher. O primeiro foi realizado para comparar a sobrevivência das vítimas ao APH do grupo que participou do estudo com os excluídos. O Teste Exato de Fisher foi utilizado na comparação dos desfechos das vítimas de PCR por causas externas e clínicas, visto que os pressupostos para aplicação do teste Qui-quadrado não foram atendidos. Para os tempos de sobrevivência, variável contínua, foram construídas curvas de sobrevivência para PCRs de natureza clínica e traumática. O teste não paramétrico log-rank foi empregado na comparação das curvas de sobrevivência, visto que o teste Shapiro-Wilk rejeitou a hipótese nula (H0) de tempo de sobrevivência com distribuição normal (p < 0,001).

### Aspectos éticos

Esta pesquisa seguiu a Resolução nº 466, de 12 de dezembro de 2012, do Plenário do Conselho Nacional de Saúde, sobre pesquisa envolvendo seres humanos, e foi previamente submetida à avaliação pelo Comitê de Ética em Pesquisa (CEP) da Escola de Enfermagem da Universidade de São Paulo, parecer n° 2.542.877, de 14 de março de 2018. A coleta de dados foi iniciada somente após aprovação.

A pesquisa obteve também anuência dos serviços envolvidos para sua realização. O termo de compromisso para utilização de informações de prontuários em projeto de pesquisa foi firmado pela pesquisadora e apresentado ao Comitê de Ética e Pesquisa das instituições.

Os pacientes que participaram da terceira fase, o fizeram mediante consentimento por meio da assinatura do TCLE. Para aqueles que não tinham condições de decidir sobre a anuência de participar da investigação, o TCLE foi aplicado aos familiares que participaram da pesquisa.

## Resultados

Excluídos os menores de 18 anos, gestantes e institucionalizados, foram selecionadas 1.051 fichas de atendimentos. Dessas, foram excluídas 161 (15,32%) ilegíveis ou incompletas e 38 (3,625) relativas a pacientes transferidos para hospitais que não participavam deste estudo. Restaram, portanto, 852 (81,06%) fichas de vítimas de PCR extra-hospitalar que compuseram a casuística desta pesquisa. Vale explicitar que a sobrevida ao APH foi similar entre os pacientes participantes ou não (com fichas ilegíveis, incompletas e dos transferidos para outros hospitais) no estudo, p=0,917, valor calculado utilizando o teste Qui-Quadrado de Pearson.

A Tabela 1 apresenta o perfil dos pacientes incluídos na pesquisa, de acordo com as variáveis sexo, faixa etária e presença de comorbidades ou hábitos de risco e a Tabela 2 mostra a frequência de comorbidades e hábitos de risco verificados. Quanto a característica dos participantes deste estudo, predominaram sexo masculino (65,26%), com média de idade de 64,33 (DP=17,16) anos. As comorbidades relatadas mais frequentemente foram hipertensão arterial (44,25%), cardiopatias (25,94%), diabetes (24,06%) e neuropatias (12,21%). Nos registros, 252 casos (29,58%) não apresentaram relato de comorbidades ou hábitos de risco.

**Tabela 1:** – Pacientes que apresentaram PCR extra-hospitalar (no=852) segundo sexo, faixa etária, comorbidades e hábitos de risco. Campo Grande (MS), 2016/2018

Variáveis	n^**o**^	%
**Sexo**		
Masculino	556	65,26
Feminino	296	34,74
**Faixa etária (anos)**		
≥18 <35	56	6,57
≥35 <50	105	12,32
≥50 <65	225	26,41
≥65 <80	312	36,62
≥ 80	154	18,08
**Comorbidades e Hábitos de Risco**		
Sim	600	70,42
Não	252	29,58

**Tabela 2:** – Frequência que os pacientes que apresentaram PCR extra-hospitalar (no=852) relataram comorbidades e hábitos de risco. Campo Grande (MS), 2016/2018

Comorbidades e hábitos de risco	n^**o**^	%
Hipertensão arterial	377	44,25
Cardiopatias	221	25,94
Diabetes	205	24,06
Neuropatias	104	12,21
Pneumopatias	57	6,69
Etilismo	45	5,28
Tabagismo	42	4,93
Câncer	38	4,46
Nefropatias	34	3,99
Doenças psiquiátricas	18	2,11
Obesidade	16	1,88
Doenças vasculares	8	0,94
Hepatopatias	8	0,94
Drogadição	6	0,70
Outras comorbidades*	6	0,70

*: artrite, lúpus, Vírus da Imunodeficiência Humana (HIV), doença de Chagas, osteoporose.

A maioria dos eventos de PCR foi de natureza clínica (89,44%) e ocorreu no domicílio (80,87%). O tempo de resposta médio até a chegada do primeiro atendimento foi de 13,37 (DP=7,35) minutos; até a chegada do SAV, foi de 19,25 (DP=10,85) minutos.

As PCR foram presenciadas em 30,87% dos casos, porém houve muitas fichas sem registro dessa informação (45,54%). Em 80,17% dos eventos, a RCP foi iniciada pela equipe de Suporte Básico de Vida ou expectadores. Em 73,35% dos atendimentos, o primeiro ritmo detectado foi não chocável, e a duração média da RCP foi de 30,17 (DP=14,59) minutos. Após a primeira PCR, 29,93% dos pacientes apresentaram RCE e 15,14% tiveram recidiva de PCR ainda no pré-hospitalar.

A Tabela 3 apresenta o desfecho dos pacientes até alta hospitalar.

**Tabela 3:** – Pacientes que apresentaram PCR extra-hospitalar (n=852) segundo desfechos do atendimento do pré e intra-hospitalar. Campo Grande, MS, Brasil, 2018/2020

Desfechos	N	%
Óbito pré-hospitalar	676	79,34
Óbito intra-hospitalar	140	16,43
Alta hospitalar	32	3,76
Transferidos	4	0,47
**Total**	852	100,00

**Figura 1:**
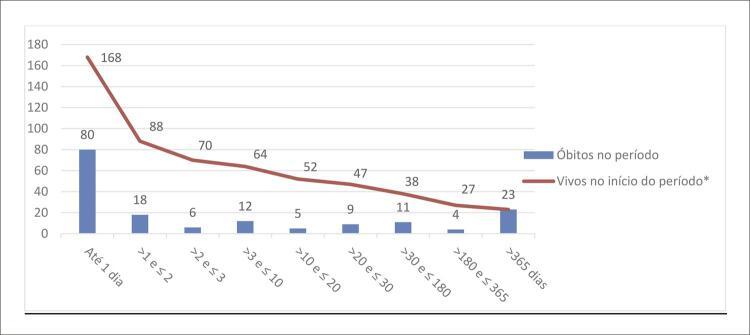
– Pacientes hospitalizados após PCR extra-hospitalar (n=168*) segundo tempo de sobrevida em dias e número de sobreviventes e óbitos no período. Campo Grande, MS, Brasil, 2018/2020. *Exclui 8 pacientes sem informação.

Já a Figura 1 mostra o tempo de sobrevida em dias e número de sobreviventes. Entre os 176 hospitalizados, houve perda de seguimento de 8 (4,55%) participantes. Dos 168 pacientes que restaram 80 (47,62%) morreram até o primeiro dia após PCR.

Nos sobreviventes à hospitalização, foi aplicado a escala de CPC em três momentos (alta, seis meses e um ano após a PCR), conforme é demonstrado na Tabela 4. Em todos os períodos de avaliação, 58,33% apresentaram desfechos favoráveis (CPC 1 e 2).

**Tabela 4:** – Pacientes que sobreviveram a internação hospitalar (n=36) conforme condições neurológicas na alta, aos seis meses e um ano após PCR, segundo Categoria de Performance Cerebral. Campo Grande, MS, Brasil, 2018/2020

Categoria de Performance Cerebral	Alta/Transferência		Após 6 meses		Após 1 ano	
	N	%	N	%	N	%
1 (Bom desempenho cerebral)	10	27,78	12	33,33	15	41,67
2 (Incapacidade cerebral moderada)	11	30,55	9	25,00	6	16,66
3 (Incapacidade cerebral grave)	5	13,89	4	11,11	1	2,78
4 (Comatoso, estado vegetativo)	2	5,56	2	5,56	1	2,78
5 (Morte)			1	2,78	5	13,89
Perdas de seguimento	8	22,22	8	22,22	8	22,22
**Total**	36	100,0	36	100,0	36	100,0

Em relação à natureza das chamadas do APH, 89,44% foram motivadas por causas clínicas. Os demais casos foram causas externas (10,56%) de diferentes mecanismos: contusos (7,39%), penetrantes (1,64%) ou outros (1,53%).

**Figura 2:**
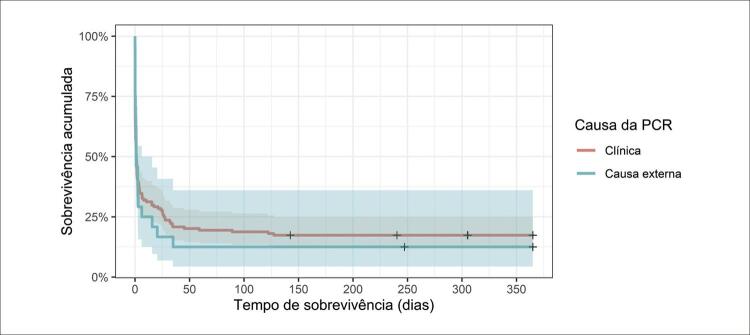
– Curvas de sobrevivência dos pacientes hospitalizados após PCR extra-hospitalar (n=176), para PCR de causa clínica e traumática. Campo Grande, MS, Brasil, 2018/2020.

Houve associação entre os desfechos observados após PCR extra-hospitalar e causa da ocorrência (p= 0,026). Nota-se na Tabela 5 que o óbito no pré-hospitalar foi mais frequente nas PCRs de causa clínica, e as mortes durante a hospitalização, nas de causas externas. Quando analisado o tempo de sobrevivência, as curvas (Figura 2) mostram tempo de sobrevida ligeiramente maior para PCR de natureza clínica, após as primeiras horas, entretanto as diferenças observadas entre os grupos não alcançaram significância estatística, segundo o teste log-rank (p=0,6).

**Tabela 5:** – Pacientes que apresentaram PCR extra-hospitalar (n=852), de acordo com a natureza da PCR, segundo desfechos no pré e intra-hospitalar. Campo Grande, MS, Brasil, 2018/2020

Desfechos	Clínicas		Causa externa		Valor p
	N	%	N	%	
Óbito pré-hospitalar	612	80,31	64	71,11	0,026*
Óbito intra-hospitalar	119	15,62	21	23,34	
Alta hospitalar	29	3,81	3	3,33	
Transferidos	2	0,26	2	2,22	
**Total**	762	100,0	90	100,0	

*Teste exato de Fisher.

## Discussão

Um dos primeiros indicadores de sucesso na ressuscitação é o RCE, que apresentou nesta pesquisa frequência de 29,93%. Há grande variação desse resultado em estudos recentes realizados em diferentes países, de 5,7% a 33%.^7-9^ Analisando apenas PCR por causas traumáticas, pesquisadores holandeses encontraram taxa de 28,5% de RCE em serviço de emergência médica com helicópteros.^10^

No Brasil, pesquisas recentes evidenciaram sobrevivência variando de 5,84% até 15,5%. Trata-se, porém, de estudos com pequenas amostras, sem informações sobre a sobrevivência à internação e após a alta hospitalar.^11,12^

A sobrevivência até a admissão hospitalar também é um resultado inicial da RCP que apresenta consideráveis variações em publicações recentes de diferentes países, com frequências de 4,4% até 33,1%.^7,13-16^

Outro importante indicador de qualidade das manobras de RCP é a sobrevivência até a alta hospitalar. Mais uma vez os resultados da literatura foram bastante diversificados, com frequências de sobrevivência de 1,6% a 31,3%.^7,8,10,13-18^ A maior frequência de sobrevida hospitalar foi observada em estudo realizado em hospitais americanos, com valor médio de 31,3% e taxas de 12,5% a 46,7% em diferentes hospitais.^18^

Em relação a essas grandes diferenças entre as taxas de RCE e sobrevivência até admissão hospitalar e alta, há de se considerar que podem decorrer tanto da qualidade do atendimento como dos critérios de inclusão dos pacientes nos estudos, características das amostras, estrutura do APH e hospitalar, critérios para iniciar e manter a RCP ou de um conjunto de fatores locais que podem modificar esses resultados. A análise de populações específicas, como casos de PCR de origem cardíaca, em ritmos chocáveis ou testemunhadas, podem trazer melhores resultados.^13^ Por outro lado, o emprego indiscriminado da RCP contribui para as estatísticas que mostram elevada frequência de insucessos, prejudicando a avaliação da sua eficácia.

O RCE e a sobrevivência até a admissão hospitalar alcançaram no atual estudo valores próximos aos mais elevados observados na literatura recente,^7-13^ no entanto a taxa de sobrevida à hospitalização foi uma das menores entre as pesquisas analisadas.^7-9,13-18^

As modalidades de APH são muito variáveis ao redor do mundo, e os melhores resultados observados nesta investigação podem estar relacionados ao atendimento do SAV para todos os participantes deste estudo, já que este conta obrigatoriamente com um médico e um enfermeiro entre os tripulantes da unidade.

As melhores condições dos pacientes na internação, em virtude da atuação do APH ou de critérios de iniciar e manter a RCP, podem melhorar os resultados no hospital, no entanto é inegável a importância da assistência hospitalar para a sobrevida.

Autores americanos analisando resultados da PCR em diferentes hospitais verificaram que as taxas de sobrevida hospitalar e o desfecho neurológico favorável variaram a depender do hospital para o qual o paciente foi transportado após a PCR e essa variação nem sempre foi explicada pelas características dos pacientes.^18^ Esses resultados sugerem que parte dos hospitais participantes do estudo apresentavam necessidade de melhoria da qualidade do atendimento para melhorar os desfechos dos pacientes após PCR.

Nos pacientes que tiveram alta hospitalar, aplicou-se o índice de CPC na alta, aos seis meses e um ano após a PCR e mais da metade dos indivíduos apresentaram desfechos favoráveis (CPC 1 e 2), em todos os períodos de avaliação. Na alta, 21 dos 28 pacientes que foram avaliados utilizando o CPC apresentaram escores de 1 e 2, resultado corroborado pela literatura recente: 1,3% dos casos com desfecho favorável em uma taxa de sobrevivência de 1,6%^7^; 4,9%, em 5,9%^9^ e 25%, em 31,3% sobreviventes à alta.^18^

Na China, estudo que analisou 5016 PCR extra hospitalares mostrou que um ano após alta hospitalar 44 (0,87%) pacientes estavam vivos e 37 (0,73%) encontravam-se em boas condições neurológicas.^7^ No atual estudo, cinco de 28 pacientes com seguimento morreram entre alta e um ano após PCR. Nesse último período, porém, somente dois pacientes apresentavam condições neurológicas desfavoráveis (CPC 3 e 4).

Estudo brasileiro com 285 pacientes atendidos com PCR em serviço de emergência encontrou, que após seis meses de seguimento, 53,8% permaneceram com o mesmo CPC, e 46,2% tiveram melhora da CPC em relação à alta. Após um ano, a totalidade dos pacientes permaneceu com a mesma CPC em relação aos seis meses anteriores.^19^

Em nossos dados, observou-se melhora das condições funcionais dos pacientes até um ano após PCR: cinco pacientes que apresentaram CPC 2 na alta alcançaram escore 1 no índice e três com CPC 3 evoluíram para pontuação 2.

O óbito no pré-hospitalar foi menos frequente nos eventos de natureza traumática no presente estudo, ao passo que as mortes durante a hospitalização ocorreram em maior frequência nesse grupo. Os percentuais de sobrevida à hospitalização foram semelhantes (3,81% causas clínicas e 3,33% causas externas), assim como o tempo de sobrevivência dos dois grupos.

Dados do Registro Francês de PCR extra-hospitalar,^20^ mostraram 12,2% dos eventos como de origem traumática e percentual de sobreviventes entre os pacientes com PCR de natureza clínica de 5,4% e de causas traumáticas, 1,7%.

Tendo em vista que esta pesquisa e vários estudos recentes que analisam sobrevida hospitalar apresentaram taxas inferiores a 5%,^7,9,13,20^ investigações com populações de PCR por causa traumática não mostraram resultados discrepantes em relação a essas publicações com taxas de sobrevivência até a alta de 3,9%^10^ e 18,6%.^17^

Em um estudo que comparou a sobrevivência à admissão e à alta hospitalar em PCR por causa traumática e não traumática, ambos os desfechos foram significativamente mais frequentes no grupo das causas não traumáticas. Porém, os autores verificaram que houve diferenças nas características dos grupos; por exemplo, as paradas cardíacas traumáticas foram menos propensas a ser testemunhadas, sendo difícil, assim, atribuir a causalidade nos resultados.^16^

Em revisão de literatura sobre PCR traumática, autores apontam que os avanços no controle de danos na RCP e na compreensão das diferenças fisiopatológicas desse evento perante os de causas clínicas levaram a sobreviventes inesperados. Dados sugerem que o desfecho da PCR traumática não é pior que o de causas clínicas e, em alguns grupos, pode até apresentar melhores resultados.^21^

Em análise de registros de PCR de 20 anos, a taxa de sobrevivência de 30 dias dobrou no período para o grupo de PCR extra-hospitalar por etiologia médica, de 4,7% para 11,0%. No grupo de causas não médicas, essa taxa triplicou, evoluindo de 3% para 9,9%. O trauma foi a causa mais comum nesse último grupo, atingindo 26% dos casos.^22^

Na Dinamarca, pesquisadores verificaram que a sobrevivência pré-hospitalar foi maior no grupo das causas médicas, porém, a sobrevivência de 30 dias e um ano foi similar entre os grupos.^23^

Diferentes classificações que incluem vítimas de trauma dificultam comparações entre os estudos, porém há evidências de que a natureza da PCR nem sempre estabelece sobrevida. Os dados demonstram que uma gama de variáveis deve ser considerada ao tentar definir prognóstico em PCR extra-hospitalar.

Considerando nossos resultados e a literatura disponível, pode-se afirmar que, apesar das diferenças ainda presentes quanto aos desfechos da PCR traumática, não há evidências para que ocorram, *a priori*, restrições para ressuscitar vítimas desse evento. Crenças sobre a futilidade da RCP em casos de trauma prejudicam a obtenção de informações seguras sobre seus desfechos e podem atrasar o aprimoramento das manobras de atendimento dessas vítimas, as quais poderiam se beneficiar com tratamentos específicos para esse grupo.

Conhecer características e desfechos da PCR extra-hospitalar pode auxiliar os gestores no planejamento de políticas de saúde, dimensionamento de equipes e gestão dos recursos públicos destinados à estruturação dos sistemas de atendimento. Este estudo também propicia estabelecer metas de melhores resultados e de reparo nas condições locais.

No âmbito da pesquisa cientifica, este é um dos primeiros trabalhos dessa magnitude realizados na cidade de Campo Grande e um dos poucos no Brasil com essa abordagem. Além de permitir comparações com futuros resultados e fornecer estatísticas brasileiras, tão escassas, pode contribuir para a formulação das diretrizes de reanimação e tratamento no país.

Autores relatam dificuldades e limitações na coleta de dados sobre as ocorrências de PCR, principalmente porque as pesquisas são retrospectivas, em sua maioria utilizando dados de eventos passados registrados.^24^

Entre as limitações desta investigação, vale salientar a dificuldade de coleta de dados, uma vez que uma fonte importante de informações são as fichas de registro de APH, as quais, muitas vezes, não são completamente preenchidas devido a premência de outras atividades nas emergências. Além disso, assim como todos os estudos de coorte, houve perdas de seguimento dos participantes.

## Conclusão

Neste estudo, a sobrevivência à hospitalização após PCR extra-hospitalar foi baixa, porém, a maioria dos sobreviventes à alta hospitalar alcançaram desfecho favorável após um ano desse evento. Entre os hospitalizados, não houve diferença no tempo de sobrevivência entre os pacientes de PCR de natureza clínica e traumática; no entanto, a sobrevivência até a hospitalização foi maior entre as PCR por causa traumática.
