# Supplementary material for: Desfechos após Parada Cardiorrespiratória Extra-Hospitalar de Natureza Clínica e Traumática
Source: Arq Bras Cardiol. 2023 Jul 19;120(7):e20220551. [Article in Portuguese] doi: 10.36660/abc.20220551 (PMC10374265; doi:10.36660/abc.20220551)
Supplement: Translation

# Translation: Outcomes after Clinical and Traumatic Out-of-Hospital Cardiac Arrest

**Authors:** Daiana Terra Nacer, Regina Márcia Cardoso de Sousa, Anna Leticia Miranda

**Affiliations:** 1 Brazil Universidade de São Paulo – Escola de Enfermagem, São Paulo, SP – Brazil; 2 Brazil Universidade Federal de Minas Gerais – Faculdade de Medicina – Campus Saúde, Belo Horizonte, MG – Brazil

**Keywords:** Heart Arrest, Out-of-Hospital Cardiac Arrest, Cardiopulmonary Resuscitation, Emergency Medical Services, Survivorship

## Abstract

**Background:**

Data on out-of-hospital cardiac arrest are still scarce, very varied, and indicate a poor prognosis for traumatic events.

**Objectives:**

To describe the out-of-hospital/in-hospital survival, survival time, and neurological conditions of those treated by advanced life support units and submitted to cardiopulmonary resuscitation and compare the results of clinical and traumatic cardiac arrests.

**Methods:**

This is a cohort study carried out in three stages; in the first two, data were collected from the Mobile Emergency Care Service forms and medical records; then, the Brain Performance Category Scale was applied in the third stage. The sample consisted of resuscitated victims aged ≥18 years. Fisher’s and log-rank tests were used to compare the causes, considering a significance level of 5%.

**Results:**

852 patients were analyzed; 20.66% were hospitalized, 4.23% survived until transfer or discharge, and 58.33% had a favorable outcome one year after arrest. There was an association between pre/in-hospital survival and the nature of the occurrence (p=0.026), but there was no difference between the survival curves (p=0.6).

**Conclusions:**

Survival of hospitalization after out-of-hospital cardiac arrest was low; however, most who survived to be discharged achieved a favorable outcome after one year. The survival time of those hospitalized after clinical and traumatic events were similar, but pre-hospital survival was higher among trauma patients.

**Central Illustration:** : Outcomes after Clinical and Traumatic Out-of-Hospital Cardiac Arrest

## Introduction

Cardiac arrest (CA) is the abrupt loss of cardiac function.^1^ It is a highly prevalent event with high morbidity and mortality.^2^ Despite advances in care, the survival of these events is low, especially in an out-of-hospital environment. Data from the literature are still scarce in Brazil, very varied worldwide, and indicate a poor prognosis for traumatic CA.^2^

According to the Brazilian Society of Cardiology, the survival of traumatic CA is around 0 to 2.6%, with cardiopulmonary resuscitation (CPR) efforts being considered futile in many studies.^2^ The 2015 European resuscitation guidelines encourage further studies on traumatic CA, as considerable survival variation is reported in the scientific literature (ranging from 0 to 27%), reflecting the heterogeneity in reported cases and the uneven care provided in different systems.^3^

Current statistics on out-of-hospital CA generally show significant geographic variations in the outcomes of these events. Some places with very poor results and others that reach important survival frequencies are noteworthy, likely consequences of efforts to optimize the effectiveness of the local survival chain obtained by identifying and adjusting its weak links.^4^

Different outcomes in analyzes of the quality of CA care have been valued, such as the return to spontaneous circulation (ROSC), survival until hospitalization and hospital discharge, and neurological condition in the short and medium term. Recovery from anoxic brain injury in patients with ROSC after CA is variable, and a range of neurological sequelae can ensue, from complete recovery to coma with brain death. Thus, the ideal CA outcome assessment should incorporate functional and neurological status.^5^

This study is justified due to the relevance of the topic presented and the lack of data on survival and short-term and medical neurological outcome of people who had out-of-hospital CA. Its objectives are to describe the out-of-hospital/in-hospital survival, survival time, and neurological conditions of those assisted by advanced life support (ALS) units and submitted to CPR and compare the results of clinical and traumatic CA.

## Method

### Study design

This regional cohort study was conducted in Campo Grande, Mato Grosso do Sul (MS), Brazil, and developed in three stages. First, retrospective data collection was performed in the first two stages, with the sources being the pre-hospital care (PC) records in the first stage and the medical records of patients who survived the PC referred to hospital units in the second. Then, the hospitalization survivors or their families were interviewed in the third stage, where the data collection was prospective.

### Data collection location

Data from the first stage were collected at the Mobile Emergency Care Service (SAMU); the information collection in the second stage was carried out in the three hospitals that are emergency hospital ports in the Urgency and Emergency Care Network. The last phase was carried out at the homes of the hospitalization survivors.

### Period

Data collection started in May 2018 and ended in March 2020.

### Population and selection criteria

The population consisted of subjects aged 18 years or older who had out-of-hospital CA in the period from Jan 1, 2016, to Dec 31, 2018, and who received CPR maneuvers by the SAMU advanced support PC team (including cases where it was initiated by another team, bystanders or others).

Pregnant women and patients with illegible and incomplete records were excluded, meaning those who did not allow access to the description of more than 50% of the clinical variables of the research. The records of cases transferred to hospitals not qualified as hospital ports of the Emergency and Urgencies Care Network of Campo Grande were also excluded.

### Information collection instruments

Data were collected by filling in two forms prepared by the researchers: the first instrument enabled transcribing PC information based on data available in the PC forms of SAV SAMU (physician and nurse form). In-hospital care information was collected from medical records and recorded in this first form. The second instrument included information about patients’ neurological conditions at discharge, at six months, and one year after the cardiac respiratory arrest, which was collected during home visits to patients who survived hospitalization.

Next, the Cerebral Performance Category Scale (CPC) was applied to assess the neurological condition of patients surviving hospitalization, as the *Sociedade Brasileira de Cardiologia*^6^ recommended. This scoring system enables assessing functional capacity after CA based on interviews with the family and recorded information, indicating the CPC scores at discharge, six months, and one year. The results were presented using the five categories of the scale: CPC 1 (Good brain performance); CPC 2 (Moderate Brain Disability); CPC 3 (Severe Brain Disability); CPC 4 (Comatose, vegetative state); and CPC 5 (Death). These categories of the analyzes were also dichotomized into favorable (CPC 1 and 2) and unfavorable (CPC 3, 4, and 5).

### Data collection

All care records provided by SAMU ALS units from 2016 to 2018 were consulted in the first data collection phase, and the CA records were manually separated. Records of people under 18 years of age, pregnant women, institutionalized and incomplete records (less than 50% of the clinical variables of the study filled out) were excluded, in addition to those transferred to hospitals that did not participate in the study. Information regarding ambulance activation times and displacements was collected from the SAMU Regulation Center electronic system.

Data in the second phase were collected from the medical records of patients referred to the three hospitals in the Emergency Care Network in Campo Grande. Thus, the hospitalization outcome was verified in this stage, and the CPC scale was applied based on medical records.

Patients who survived hospitalization or their family members were invited to participate in the study through a telephone call in the third phase. After the participants’ consent, they were visited at home to collect data through an interview and sign the Informed Consent Form (ICF).

All interviews in this study phase were conducted at least one year after CA, and if necessary, a caregiver/responsible person was established to provide the information for those patients unable to communicate. The CPC score obtained from the analysis of the medical records was validated during the interview in this phase, and the information about the patients’ neurological conditions at six months and one year after CA was questioned to establish the CPC scores in these last two periods.

### Data treatment and analysis

The collected data were stored in a Microsoft Office Excel^®^ database, version 2016, and this program was also used to perform the descriptive analyses. Statistical tests were performed according to guidance from a professional in the area, and the statistical package R version 4.1.0 was used, considering a significance level of 5%.

Categorical variables were described using absolute and relative frequencies, continuous variables were presented as intervals, and the mean and standard deviation (SD) were calculated in cases of normal data distribution.

When comparing the outcomes of victims of clinical and traumatic CA, the vital condition of the victims until hospitalization and discharge (categorical variables) and survival time in days after CA (continuous variable) were analyzed as dependent variables. The nature of CA (clinical or traumatic) was an independent categorical variable for these analyzes.

Pearson’s chi-squared and Fisher’s exact tests were applied to assess the association between categorical variables. The first was to compare the survival of the victims to the PC of the group that participated in the study with the excluded victims. Fisher’s Exact Test was used to compare the outcomes of CA victims due to external and clinical causes since the assumptions for applying the Chi-squared test were not met. Survival times constituted a continuous variable; survival curves were constructed for clinical and traumatic CAs. The non-parametric log-rank test was used to compare the survival curves since the Shapiro-Wilk test rejected the null hypothesis (H_0_) of survival time with a normal distribution (p < 0.001).

### Ethical aspects

This study followed Resolution no. 466, of Dec 12, 2012, of the Plenary of the National Health Council, on research involving human beings and was previously submitted for evaluation by the Research Ethics Committee (*CEP*) of the School of Nursing of the University of São Paulo, opinion No. 2,542,877, of Mar 14, 2018. Data collection was started only after approval.

The study also obtained consent from the services involved for its performance. The term of commitment for using information from medical records in a research project was signed by the researcher and presented to the Ethics and Research Committee of the institutions.

Patients who participated in the third phase did so with consent by signing the ICF. For those unable to decide whether to consent to participate in the investigation, the ICF was applied to the family members who participated in the study.

## Results

In excluding those under 18, pregnant women, and those institutionalized, 1,051 attendance records were selected. Of these, 161 (15.32%) were illegible or incomplete, and 38 (3.625) were related to patients transferred to hospitals that did not participate in this study. Therefore, there were 852 (81.06%) records of victims of out-of-hospital CA, which composed the sample of this study. It is worth noting that PC survival was similar between participating and non-participating patients (with illegible and incomplete records and those transferred to other hospitals) in the study (p=0.917), the value calculated using Pearson’s Chi-Squared test.

Table 1 presents the profile of the patients included in the study according to the variables: gender, age group, and presence of comorbidities or at-risk habits, and Table 2 shows the frequency of comorbidities and at-risk habits verified.

**Table 1:** – Patients with out-of-hospital CA (no=852) according to gender, age group, comorbidities, and at-risk habits. Campo Grande (MS), Brazil, 2016/2018

Variables	No.	%
**Gender**		
Male	556	65.26
Female	296	34.74
**Age range (years)**		
≥18 <35	56	6.57
≥35 <50	105	12.32
≥50 <65	225	26.41
≥65 <80	312	36.62
≥ 80	154	18.08
**Comorbidities and at-risk habits**		
Yes	600	70.42
No	252	29.58

**Table 2:** – Frequency that patients with out-of-hospital CA (no=852) reported comorbidities and at-risk habits. Campo Grande (MS), Brazil, 2016/2018

Comorbidities and at-risk habits	No.	%
Arterial hypertension	377	44.25
Cardiopathies	221	25.94
Diabetes	205	24.06
Neuropathies	104	12.21
Pneumopathies	57	6.69
Alcoholism	45	5.28
Smoking	42	4.93
Cancer	38	4.46
Nephropathies	34	3.99
Psychiatric illnesses	18	2.11
Obesity	16	1.88
Vascular diseases	8	0.94
Hepatopathies	8	0.94
Drug addiction	6	0.70
Other comorbidities*	6	0.70

*: arthritis, lupus, Human Immunodeficiency Virus (HIV), Chagas disease, osteoporosis.

Regarding the characteristics of the participants in this study, there was a predominance of males (65.26%), with a mean age of 64.33 (SD=17.16) years. The most frequently reported comorbidities were high blood pressure (44.25%), heart disease (25.94%), diabetes (24.06%), and neuropathies (12.21%). A total of 252 cases (29.58%) in the records had no reports of comorbidities or at-risk habits.

Most CA events were clinical in nature (89.44%) and occurred at home (80.87%). The average response time until the arrival of the first service was 13.37 (SD=7.35) minutes; it was 19.25 (SD=10.85) minutes until the ALS arrived.

CAs were witnessed in 30.87% of cases, but many files were without recording this information (45.54%). CPR was initiated by the Basic Life Support team or bystanders in 80.17% of the events. The first rhythm detected in 73.35% of cases was non-shockable, and the mean duration of CPR was 30.17 (SD=14.59) minutes. After the first CA, 29.93% of the patients had ROSC, and 15.14% had CA recurrence, even in the pre-hospital setting.

Table 3 shows the outcome of patients until hospital discharge.

**Table 3:** – Patients with out-of-hospital CA (n=852) according to pre- and in-hospital care outcomes. Campo Grande, MS, Brazil, 2018/2020.

Outcomes	N	%
Pre-hospital death	676	79.34
In-hospital death	140	16.43
Hospital discharge	32	3.76
Transferred	4	0.47
**Total**	852	100.00

Figure 1 shows the survival time in days and the number of survivors. Among the 176 hospitalized, 8 (4.55%) participants were lost to follow-up. Of the 168 remaining patients, 80 (47.62%) died within the first day after CA.

**Figure 1:** – Patients hospitalized after out-of-hospital CA (n=168*) according to survival time in days and the number of survivors and deaths in the period. Campo Grande, MS, Brazil, 2018/2020.* Excluded 8 patients without information.

The CPC scale was applied to hospitalization survivors in three moments (discharge, six months, and one year after CA), as shown in Table 4. 58.33% had favorable outcomes in all evaluation periods (CPC 1 and 2).

**Table 4:** – Patients who survived hospitalization (n=36) according to neurological conditions at discharge, at six months, and one year after CA, according to Cerebral Performance Category. Campo Grande, MS, Brazil, 2018/2020

Brain Performance Category	Discharge/Transfer		After 6 months		After 1 year	
	N	%	N	%	N	%
1 (Good brain performance)	10	27.78	12	33.33	15	41.67
2 (Moderate brain impairment)	11	30.55	9	25.00	6	16.66
3 (Severe brain impairment)	5	13.89	4	11.11	1	2.78
4 (Comatose, vegetative state)	2	5.56	2	5.56	1	2.78
5 (Death)			1	2.78	5	13.89
Follow-up losses	8	22.22	8	22.22	8	22.22
**Total**	36	100.0	36	100.0	36	100.0

Regarding the nature of PC calls, 89.44% were motivated by clinical causes. The remaining cases were external causes (10.56%) of different mechanisms: blunt (7.39%), penetrating (1.64%), or other (1.53%).

There was an association between the outcomes observed after out-of-hospital CA and the cause of occurrence (p= 0.026). It is noted in Table 5 that pre-hospital death was more frequent in clinical CAs and deaths during hospitalization in those of external causes. When analyzing the survival time, the curves (Figure 2) show a slightly longer survival time for clinical CA after the first hours; however, the differences observed between the groups did not reach statistical significance according to the log-rank test (p= 0.6).

**Table 5:** – Patients with out-of-hospital CA (n=852) according to the nature of the CA and according to pre- and in-hospital outcomes. Campo Grande, MS, Brazil, 2018/2020

Outcomes	Clinical		External cause		p-value
	N	%	N	%	
Pre-hospital death	612	80.31	64	71.11	0.026*
In-hospital death	119	15.62	21	23.34	
Hospital discharge	29	3.81	3	3.33	
Transferred	2	0.26	2	2.22	
**Total**	762	100.0	90	100.0	

*Fisher’s Exact Test.

**Figure 2:** – Survival curves of hospitalized patients after out-of-hospital CA (n=176) for clinical and traumatic CA. Campo Grande, MS, Brazil, 2018/2020.

## Discussion

One of the first indicators of success in resuscitation is ROSC, which presented a frequency of 29.93% in this study. This result is greatly varied in recent studies in different countries, from 5.7% to 33%.^7-9^ In only analyzing CA due to traumatic causes, Dutch researchers found a rate of 28.5% of ROSC in medical emergency services with helicopters.^10^

Recent studies in Brazil have shown survival ranging from 5.84% to 15.5%. However, these are studies with small samples, without information on survival after hospitalization and after hospital discharge.^11,12^ Survival until hospital admission is also an initial result of CPR which shows considerable variations in recent publications from different countries, with frequencies from 4.4% to 33.1%.^7,13-16^

Another important indicator of the quality of CPR maneuvers is survival to hospital discharge. Once again, the results in the literature were quite diverse, with survival rates from 1.6% to 31.3%.^7,8,10,13-18^The highest hospital survival frequency was observed in a study carried out in American hospitals, with a mean value of 31.3% and rates from 12.5% to 46.7% in different hospitals.^18^

Regarding these large differences between ROSC and survival rates until hospital admission and discharge, it must be considered that they may be due to both the care quality and the inclusion criteria of patients in the studies, characteristics of the samples, the PC structure, and the hospital itself, criteria for initiating and maintaining CPR, or a set of local factors which may modify these outcomes. Analyzing specific populations, such as CA cases of cardiac origin, in shockable rhythms or being witnessed, can bring better results.^13^ On the other hand, the indiscriminate use of CPR contributes to the statistics that show a high frequency of failures, undermining the evaluation of its effectiveness.

ROSC and survival until hospital admission in the current study reached values close to the highest observed in recent literature,^7-13^ however, the hospitalization survival rate was one of the lowest among the analyzed studies.^7-9,13-18^

PC modalities are very variable around the world, and the best results observed in this investigation may be related to ALS care for all participants in this study since it is mandatory to have a doctor and a nurse among the unit’s crew. The better conditions of patients during hospitalization due to the PC’s performance or criteria for initiating and maintaining CPR can improve the in-hospital results; however, the importance of hospital care for survival is undeniable.

American authors analyzing CA results in different hospitals found that hospital survival rates and favorable neurological outcomes varied depending on the hospital to which the patient was transported after CA, and the patient’s characteristics^18^ did not always explain this variation. These results suggest that part of the hospitals participating in the study needed to improve the care quality to improve patient outcomes after CA.

The CPC index was applied to patients discharged from the hospital at the discharge time, at six months, and one year after CA, and more than half of the individuals had favorable outcomes (CPC 1 and 2) in all evaluation periods. At discharge, 21 of the 28 patients who were evaluated using the CPC had scores of 1 and 2, corroborating a result in recent literature: 1.3% of cases with a favorable outcome with a survival rate of 1.6%^7^; 4.9% in 5.9%^9^; and 25% in 31.3% who survived discharge.^18^

A study in China that analyzed 5,016 out-of-hospital CAs showed that 44 (0.87%) patients were alive one year after hospital discharge, and 37 (0.73%) were in good neurological condition.^7^ In the current study, five out of 28 patients under follow-up died between discharge and one year after CA; however, only two patients had unfavorable neurological conditions (CPC 3 and 4) in this last period.

A Brazilian study with 285 patients treated with CA in an emergency service found that 53.8% remained with the same CPC after six months of follow-up, and 46.2% had an improvement in the CPC concerning discharge; moreover, all patients maintained the same CPC after one year compared to the previous six months.^19^

An improvement in the functional conditions of patients was observed in our data up to one year after CA: five patients who had CPC 2 at discharge reached a score of 1 on the index, and three with CPC 3 evolved to score 2. Pre-hospital death was less frequent in events of a traumatic nature in the present study, while deaths during hospitalization occurred more frequently in this group. The survival after hospitalization percentages were similar (3.81% clinical causes and 3.33% external causes), as well as the survival time of both groups.

Data from the French Registry for out-of-hospital CA^20^showed 12.2% of the events as having a traumatic origin, and the percentage of survivors among patients with clinical CA was 5.4%, and 1.7% for traumatic causes.

Considering that this study and several recent studies which analyzed hospital survival presented rates below 5%,^7,9,13,20^investigations with CA populations due to traumatic causes did not show discrepant results about these publications, with survival to discharge rates of 3.9%^(10)^and 18.6%.^17^

In a study that compared survival to hospital admission and hospital discharge in CA due to traumatic and non-traumatic causes, both outcomes were significantly more frequent in the group of non-traumatic causes. However, the authors found differences in the groups’ characteristics; for example, traumatic CAs were less likely to be witnessed, thus making it difficult to attribute causality to the results.^16^

In a literature review on traumatic CA, the authors noted that advances in damage control in CPR and understanding the pathophysiological differences between this event and clinical causes led to unexpected survivors. Data suggest that the outcome of traumatic CA is not worse than that of clinical causes, and in some groups, it may even present better results.^21^

In an analysis of 20-year CA records, the 30-day survival rate doubled over the period for the out-of-hospital CA group due to medical etiology, from 4.7% to 11.0%. This rate tripled in the group of non-medical causes, rising from 3% to 9.9%. Trauma was the most common cause in this last group, reaching 26% of cases.^22^ Researchers in Denmark found that pre-hospital survival was higher in the group with medical causes; however, the 30-day and one-year survival was similar between the groups.^23^

Different classifications that include trauma victims make comparisons between studies difficult, but there is evidence that the nature of CA does not always establish survival. The data demonstrate that various variables must be considered when defining prognosis in out-of-hospital CA.

Considering our results and the available literature, it can be stated that despite the differences still present regarding the outcomes of traumatic CA, there is no evidence that there are *a priori* restrictions to resuscitate victims of this event. Beliefs about the futility of CPR in trauma cases hinder obtaining reliable information about its outcomes and may delay improvement in care maneuvers for these victims, who could benefit from specific treatments for this group.

Knowing the characteristics and outcomes of out-of-hospital CA can help managers plan health policies, sizing teams, and manage public resources for structuring care systems. This study also propitiates establishing goals for better results and repair under local conditions.

Within the scope of scientific research, this is one of the first works of this magnitude carried out in Campo Grande and one of the few in Brazil with this approach. In addition to allowing comparisons with future results and providing Brazilian statistics, which are so scarce, it can contribute to formulating resuscitation and treatment guidelines in the country. Some authors have reported difficulties and limitations in collecting data on the occurrence of CA, mainly because the studies are retrospective, mostly using data from past recorded events.^24^

Among the limitations of this investigation, it is worth mentioning the difficulty in collecting data since an important information source is the PC registration forms, which are often not completely filled out due to the urgency of other activities in emergencies. In addition, as with all cohort studies, participants were lost to follow-up.

## Conclusion

In this study, survival until hospitalization after out-of-hospital CA was low; however, most survivors of hospital discharge achieved a favorable outcome after one year of this event. Among those hospitalized, there was no difference in survival time between clinical and traumatic CA patients; however, survival until hospitalization was higher among those with CA due to traumatic causes.
